# Editorial: Reviews in non-coding RNA: 2023

**DOI:** 10.3389/fgene.2024.1437522

**Published:** 2024-06-13

**Authors:** Bodhisattwa Banerjee, Sandip Mukherjee

**Affiliations:** ^1^ Department of Biochemistry, Larner College of Medicine, University of Vermont, Burlington, United States; ^2^ Division of Nutritional Science & Obesity Medicine, Washington University in St. Louis, St. Louis, United States

**Keywords:** non-coding RNA, osteoarthiritis, M6A modification, genome editing, colorectal cancer, ovarian cancer, lncRNA—long noncoding RNA, circRNA

## Introduction

In the dynamic landscape of molecular biology, long non-coding RNAs (lncRNAs) have emerged as pivotal players, far from the “junk” DNA they were once thought to be. Recent review articles have underscored the immense importance of studying the function of lncRNAs and exploring methods to inactivate their function. These comprehensive reviews not only emphasize the pressing need for loss-of-function studies to unravel the biological roles of lncRNAs but also highlight the sophisticated methodologies available for these investigations. The urgency of this research cannot be overstated, as it holds the potential to revolutionize our understanding of gene regulation and disease progression, particularly in the realms of cancer and chronic conditions such as osteoarthritis.

## CRISPR-based genome editing: The forefront of lncRNA functional studies

Among the plethora of techniques to inactivate lncRNA function, CRISPR-based genome editing stands out as the most widely adopted. This powerful tool has enabled researchers to perform precise genetic modifications, offering two main strategies for lncRNA functional knockout: the removal of the promoter and first exon and the insertion of a pre-termination poly(A) signal. Each method has its own set of advantages and challenges. For instance, while promoter and exon removal can effectively abolish lncRNA expression, it might inadvertently affect neighboring genes. Conversely, inserting a poly(A) signal can halt transcription efficiently but might not completely eliminate lncRNA function if alternative transcription start sites are used. Understanding these nuances is crucial for designing robust experiments and interpreting results accurately (Lyu et al.).

## The genetic regulatory relationship between ncRNAs and m6A modification: a new Horizon in cancer research

The intricate genetic regulatory relationship between non-coding RNAs (ncRNAs) and N6-methyladenosine (m6A) modification opens another exciting chapter in cancer biology. m6A, the most prevalent epigenetic modification of RNA in eukaryotes, plays a significant role in controlling gene expression, particularly in cancer progression. The interplay between m6A and various ncRNAs, including microRNAs, circular RNAs, and piRNAs, is crucial for understanding the molecular underpinnings of cancer. This interaction not only affects the stability and function of ncRNAs but also modulates their ability to regulate target genes. By decoding these complex relationships, researchers can identify new diagnostic and prognostic biomarkers, offering new avenues for cancer detection and treatment (Liu and Xiang).

## lncRNAs in osteoarthritis: Biomarkers and therapeutic targets

In the context of osteoarthritis, lncRNAs have been identified as key regulatory molecules influencing disease progression. The review articles highlight the diverse functions and molecular mechanisms of specific lncRNAs, including their interactions with microRNAs (miRNAs) and target genes. These insights suggest that lncRNAs could serve as valuable biomarkers for early diagnosis and as therapeutic targets to halt or reverse disease progression. As our understanding of these molecular pathways deepens, the potential for developing novel treatments for osteoarthritis becomes increasingly tangible (Zhang et al.).

## JAK/STAT signaling and lncRNAs in colorectal cancer

The regulation of the JAK/STAT signaling pathway by lncRNAs in colorectal cancer (CRC) presents another promising research Frontier. The JAK/STAT pathway is integral to gene expression and cell growth, and its dysregulation is often linked to cancer. lncRNAs can modulate this pathway, influencing CRC development by promoting cancer cell proliferation or inhibiting tumor growth. Furthermore, lncRNAs impact immune responses, suggesting potential roles in immunotherapy for CRC. Understanding these regulatory mechanisms can lead to innovative diagnostic tools and personalized treatment strategies, potentially transforming patient outcomes (Ghasemian et al.).

## Circular RNAs and drug resistance in ovarian cancer

Another critical area of study is the role of circular RNAs (circRNAs) in drug resistance, particularly in ovarian cancer. These circRNAs are involved in various mechanisms that promote drug resistance, posing significant challenges for effective treatment. Targeting circRNAs offers a promising strategy to overcome this resistance, paving the way for more effective therapeutic interventions. By elucidating the specific actions of circRNAs in drug resistance, researchers can develop targeted therapies to improve patient survival rates and quality of life (Zhan et al.).

## BLACAT1: A prognostic biomarker in cancer

Finally, the lncRNA BLACAT1 has emerged as a significant prognostic biomarker in cancer. High expression levels of BLACAT1 are associated with poor overall survival (OS) and progression-free survival (PFS) across various cancer types. This correlation underscores BLACAT1’s potential as a valuable marker for predicting cancer prognosis and guiding treatment decisions. However, further research is essential to validate these findings and address existing limitations, ensuring that BLACAT1 can be effectively integrated into clinical practice (Yan et al.).

## Conclusion

The exploration of lncRNAs and their multifaceted roles in gene regulation, disease progression, and therapeutic resistance represents a critical Frontier in biomedical research. The insights gained from these studies not only enhance our understanding of fundamental biological processes but also hold the promise of novel diagnostic and therapeutic strategies. As we continue to decode the complex language of lncRNAs and their interactions with other molecular players, the potential to transform patient care and outcomes becomes ever more achievable. The urgency and importance of this research cannot be overstated, as it heralds a new era in precision medicine and personalized healthcare.

